# Trigeminal Neuralgia in Multiple Sclerosis: A Critical Review of the Therapeutic Evidence and the Role of Cerebellopontine Angle Surgical Exploration

**DOI:** 10.3390/brainsci16080881

**Published:** 2026-08-19

**Authors:** Luis Ley Urzaiz, Rodrigo Carrasco Moro

**Affiliations:** Department of Neurosurgery, Hospital Universitario Ramón y Cajal, 28034 Madrid, Spain

**Keywords:** trigeminal neuralgia, multiple sclerosis, surgical exploration, cerebellopontine angle, microvascular decompression, neurolysis, stereotactic radiosurgery, health-related quality of life, medication burden, patient-centered outcomes

## Abstract

Background: Trigeminal neuralgia associated with multiple sclerosis (TN-MS) is a secondary facial pain syndrome whose clinical and therapeutic features differ substantially from those of classical TN. Although the literature comparing treatment outcomes between TN-MS and classical TN is relatively abundant, evidence on the impact of treatment on quality of life in TN-MS is scarce. In patients with MS—already vulnerable owing to the course of their disease and the burden of its treatments—therapeutic success cannot be defined by pain scales alone, because even modest analgesic improvement and, in particular, a reduction in medication burden may represent meaningful gains. This review aims to reappraise the therapeutic goals in TN-MS. Methods: We provide a critical narrative review of the evidence, explicitly distinguishing direct evidence from indirect evidence extrapolated from the MS and classical-TN literature, across medical therapy, percutaneous procedures, stereotactic radiosurgery, and microvascular decompression (MVD). Results: First-line pharmacotherapy remains standard but is largely extrapolated from classical TN, and tolerability is frequently limited by sedative and motor adverse effects in already polymedicated patients. Percutaneous procedures and radiosurgery provide clinically meaningful relief in selected patients, but recurrence is common and patient-centered outcomes are poorly documented. Surgical exploration of the cerebellopontine angle—including MVD, neurolysis, and combined techniques—should not be categorically excluded on the basis of an MS diagnosis in carefully selected patients, although outcomes are generally less favorable than in classical TN and the direct evidence is limited and vulnerable to selection bias. Conclusions: Future studies should assess treatment impact on additional outcomes such as medication burden, quality of life, fatigue, cognition, and functional status.

## 1. Introduction

Trigeminal neuralgia (TN) is one of the most characteristic pain syndromes of multiple sclerosis (MS), in which it occurs far more frequently than in the general population [1,2]. Compared with classical TN, TN-MS tends to present at a younger age and is more frequently associated with features such as bilateral involvement, sensory abnormalities, and interparoxysmal pain, which reflect a distinct pathophysiological substrate and complicate the selection of optimal treatment [2,3,4,5].

The most important distinction, however, is conceptual. TN-MS should not be regarded as a TN that occurs incidentally alongside demyelinating disease, because it develops in a clinical setting of high vulnerability, typically characterized by fatigue, cognitive inefficiency or “brain fog”, mood symptoms, and exposure to multiple medications together with the demands of treatment adherence [6,7,8,9,10]. In this context, the side effects and adverse effects of the treatments received, as well as drug–drug interactions, may induce functional worsening despite improvement of the facial pain [6,7,8,9,10].

This problem has been insufficiently recognized in the TN-MS literature. Most treatment studies focus essentially on the likelihood of immediate and complete pain relief and on recurrence rates, but few report the impact of treatment on variables such as health-related quality of life, fatigue, cognition, or the reduction in medication requirements [1,2,3,11]. As a result, treatment success is commonly defined using essentially pain-based metrics that may underestimate the burden of chronic anticonvulsant polytherapy in a neurologically vulnerable population [6,7,8,9,10].

The aim of this review is therefore not only to summarize the evidence on the various treatment modalities—medical, ablative, radiosurgical, and decompressive—in TN-MS, but also to reframe the therapeutic goals in this specific clinical context.

## 2. Materials and Methods

A non-systematic search was performed in PubMed/MEDLINE using the MeSH terms “trigeminal neuralgia” AND “multiple sclerosis”, complemented by treatment-specific searches: “microvascular decompression”, “stereotactic radiosurgery”, “percutaneous rhizotomy”, “gamma knife”, and “polypharmacy”. The search period spanned January 2000 to April 2026, with additional inclusion of seminal references predating this period when their relevance justified it. Priority was given to studies reporting outcomes in exclusive or predominantly TN-MS cohorts, as well as to systematic reviews and meta-analyses. References on the impact of polypharmacy and medication burden in MS were identified through specific secondary searches. Given its critical and narrative nature, this synthesis does not follow a formal PRISMA protocol; reference selection was guided by clinical relevance and methodological quality, at the authors’ discretion.

## 3. Pathophysiology

The central pathogenic mechanism of TN-MS is the development of a focal demyelinating lesion affecting the trigeminal root entry zone (REZ) or the intrapontine trigeminal pathways, favoring ectopic impulse generation and ephaptic transmission [2,4,12]. These phenomena provide a biologically coherent explanation both for the paroxysmal, stimulus-evoked character of the pain and for the epidemiological and clinical differences between TN-MS and classical TN [2,4,12].

This pathogenic model is, however, only partial. Neurovascular conflict may coexist with a demyelinating plaque in a substantial proportion of patients with TN-MS, as shown by series studied with magnetic resonance imaging (MRI) and at surgical exploration [2,3,11,13,14,15]. The identification of neurovascular contact does not constitute irrefutable proof of pathogenicity, as it is also observed in asymptomatic individuals and in patients without a typical classical-TN syndrome [16,17]. Consequently, the main question is not whether vascular contact exists, but whether it is pathogenically relevant in a given patient.

These observations have contributed to the development of the pathogenic model known as the “double-crush” hypothesis. In TN-MS, this hypothesis proposes that a demyelinated trigeminal root becomes more susceptible to the symptomatic effects of vascular compression, such that two individually subthreshold insults together would produce neuralgia [2,3]. The model is plausible and would simplify the therapeutic heuristic regarding MVD. Nevertheless, in the absence of direct mechanistic proof, it should be regarded as expert opinion rather than an established fact [2,3].

In this respect, imaging provides structural evidence but does not fully eliminate uncertainty. Fine-slice cisternal MRI can identify neurovascular relationships, whereas standard MS protocols can detect pontine lesions at the root entry zone [2,3,16,17]. However, these techniques cannot reliably determine which lesion is dominant in symptom generation, nor robustly predict the response to a given intervention in individual patients [2,3,11]. In current practice, MRI should be used to support a model of mechanistic probability rather than to create a false sense of certainty.

## 4. Clinical Features

TN-MS typically presents one or two decades earlier than classical TN and shows a much higher frequency of bilateral involvement [2,4,5]. Sensory deficit, persistent dysesthesia, and concomitant background pain are also more common than in classical TN, supporting the view that central pathway damage is usually more extensive in TN-MS [2,3,5].

These features are clinically important because they may modify treatment expectations. A purely paroxysmal syndrome with clear triggers, minimal background pain, and limited sensory loss may indicate a phenotype closer to compression-dominant TN, especially when neurovascular conflict is demonstrated on MRI [2,3,11]. Conversely, mixed pain patterns, sensory impairment, or extensive pontine demyelination may indicate a phenotype in which no peripheral intervention is likely to work as well as it does in classical TN [2,3].

Integrated clinical and neuroradiological diagnostic discipline is therefore decisive. A diagnosis of TN-MS does not exclude a pathophysiological basis similar to the classical form, and it should reduce confidence that any single anatomical lesion fully explains the syndrome. This is one reason why treatment decisions in TN-MS should be individualized rather than based on rigid dichotomous algorithms [2,3,11].

## 5. Medical Treatment

Medical treatment remains the accepted first-line approach for TN-MS, but this position is based largely on extrapolation from classical TN rather than on direct evidence from dedicated clinical trials [1,2,3,18,19]. Carbamazepine and oxcarbazepine are used on the basis of their established role in classical TN and expert practice in secondary TN, not on the basis of dedicated placebo-controlled trials demonstrating benefit specifically in TN-MS [2,3,18,19].

This distinction is not semantic. Direct evidence on optimal dosing, efficacy, discontinuation patterns, or comparative tolerability in TN-MS is limited. Expert reviews consistently note that sodium-channel blockers remain the starting point, but also that the development of sedative and motor adverse effects frequently prompts early consideration of pharmacological alternatives or of a surgical procedure [2,3].

Adjuvant agents such as baclofen, lamotrigine, gabapentin, or pregabalin are commonly used in practice, especially when continuous pain coexists with paroxysms [2,3,20,21]. Baclofen has historical support in TN in general, and lamotrigine has limited evidence from studies in refractory TN, but neither is supported by modern, TN-MS-specific trial data [20,21]. Polytherapy with multiple neuromodulators represents a pragmatic strategy in the face of refractoriness rather than an evidence-optimized regimen. From a clinical-management perspective, this dynamic generates an adverse-management bias: refractoriness and drug intolerance prompt frequent changes to the therapeutic regimen, synergistically increasing the cumulative burden of adverse effects on an already vulnerable neurological substrate. In parallel, the assumption of lower predictive efficacy of surgical options in TN-MS often results in late referral, postponing the individualized assessment of the risk–benefit ratio and perpetuating pharmacological escalation as the only available alternative.

## 6. Percutaneous Procedures

Percutaneous procedures—including radiofrequency thermocoagulation, glycerol rhizotomy, and balloon compression—remain widely used in TN-MS because of their inherent familiarity, repeatability, and lower technical demands compared with posterior fossa surgical exploration [1,2,3]. Their role is particularly important in patients with significant disability, unfavorable operative risk, inconclusive imaging, or a clinical phenotype that does not firmly support MVD [1,2,3].

The evidence base is modest. A 2018 systematic review showed that the available literature on TN-MS treatment was dominated by retrospective series with heterogeneous outcome definitions and limited quality-of-life reporting [1]. Within these limitations, percutaneous procedures appear to provide significant short-term pain relief, but with recurrence rates generally higher than in classical TN [1,2,3]. This is unsurprising, since these procedures act on an anatomical area distal to the dominant lesion, especially when the underlying mechanism is a central demyelinating plaque without an added peripheral neurovascular conflict [2,3].

The complication profile must also be interpreted in the specific context of MS. The development of anesthesia, dysesthesia, or abolition of the corneal reflex has a more severe clinical impact in patients with pre-existing compromise of central neurological reserve [1,2,3].

## 7. Stereotactic Radiosurgery

Stereotactic radiosurgery, often delivered as Gamma Knife radiosurgery (GKRS), offers a non-invasive treatment alternative that is attractive for patients with substantial comorbidity, high anesthetic risk, or those considered frail, in order to avoid a craniotomy [1,2,3,22,23]. In TN-MS, this appeal is, if anything, even more evident [2,3].

Direct evidence in TN-MS also indicates that GKRS can provide clinically significant relief, but with shorter durability than in classical TN [22,23]. In a large institutional series, both initial and repeat GKRS were effective, although the duration of pain relief after the first treatment was shorter in TN-MS than in historical TN cohorts [22]. A 2024 systematic review and meta-analysis likewise supports GKRS as a reasonable option in TN-MS, but confirms the heterogeneous quality of the studies and of outcome reporting [23]. These data must be interpreted with caution. The available radiosurgical literature in TN-MS is predominantly retrospective and lacks systematic stratification according to lesional findings on neuroimaging [1,22,23].

## 8. Microvascular Decompression

MVD remains a central point of controversy in the management of TN-MS. The pathophysiological argument against MVD in this setting is that, if the dominant lesion is demyelination at the root entry zone or within the pons, decompression should not reproduce the excellent outcomes observed in classical TN [2,3]—a rationale that has historically led many surgeons to contraindicate this technique in patients with MS. Counter to this position, several operative series published over the past two decades, such as those of Broggi et al. and Montano et al., support a benefit of MVD in selected patients, with certain limitations [11,13,14,15]. The proportion of pain-free patients was generally lower than in classical TN and, with regard to imaging findings, neither the presence of neurovascular compression nor of a pontine demyelinating plaque was predictive of outcome [11].

The most recent quantitative evidence supports this nuanced reading. A meta-analysis of long-term outcomes of MVD in TN-MS placed the sustained pain-free rate at around 30%, concluding that the technique is particularly useful in the presence of neurovascular conflict [24]. Nonetheless, it has been argued that excessively restrictive outcome definitions—limited to complete pain freedom—may underestimate the true clinical benefit of MVD, insofar as they do not capture the transition from uncontrolled pain to pain controlled with a reduced medication burden, an outcome that is especially relevant in a population in which anticonvulsants frequently worsen the underlying neurological symptoms [25].

The literature supports a balanced position. MVD should not be promoted as a standard solution for TN-MS, because the overall evidence base is weak and has not demonstrated comparative superiority [1,2,3,11]. Equally, it should not be categorically excluded in patients diagnosed with MS [2,3,11,13,14,15]. In carefully selected patients with acceptable operative risk, a predominantly classical clinical expression, and a convincing neurovascular conflict, MVD represents a reasonable therapeutic option.

The indication for surgical exploration in TN-MS rests on two distinct pathophysiological rationales: (1) In patients with imaging-confirmed neurovascular conflict and a predominantly classical, trigger-dependent clinical phenotype, microvascular decompression aims to relieve root compression. (2) In patients without clear preoperative imaging evidence of neurovascular conflict, but with refractory pain despite multiple prior treatments and a clinical phenotype suggesting possible compressive pathology, surgical exploration may still be warranted to identify MRI-occult conflicts at intraoperative inspection or to perform intraoperative neuromonitoring-guided techniques such as root combing and internal neurolysis. Published series indicate that neither the presence of neurovascular compression nor of a demyelinating plaque on preoperative imaging reliably predicts surgical outcome in TN-MS [11], underscoring that imaging findings alone should not be used to exclude patients from surgical consideration.

In the absence of a clearly demonstrated neurovascular conflict on neuroimaging, the indication for surgical exploration should therefore be considered exceptionally, within a multidisciplinary evaluation, and with the dual purpose of identifying compressions not detected on imaging or of applying intraoperative neuromonitoring-guided techniques such as root combing and internal neurolysis.

## 9. Comparative Analysis

The greatest weakness of the TN-MS treatment literature is not only the low level of evidence, but a questionable outcome hierarchy. Regardless of the modality used, treatment success is generally defined by pain response (measured according to the corresponding Barrow Neurological Institute scale category), recurrence rate, and sensory morbidity [1,2,3,11,22,23]. These outcomes are important, but insufficient in the special case of TN-MS.

A treatment strategy that produces pain control similar to that of another alternative, but that allows a reduction in sedative medication, might be preferable to one that achieves a marginally better pain score while prolonging polytherapy. Conversely, a procedure that resolves the pain but leaves the patient with facial numbness, functionally limited, and still dependent on medication may be less successful than conventional outcome reporting suggests.

For this reason, comparative analysis in TN-MS must extend beyond conventional concepts to include other variables such as invasiveness, probable durability, tolerability burden, medication implications, and the actual availability of patient-centered outcome data. In these terms, no modality is uniformly superior. Each occupies a different position within a matrix of uncertainty, trade-offs, and mechanistic plausibility (Table 1).

## 10. Clinical Decision Framework

In the absence of a universally validated treatment sequence, current practice relies on retrospective data and expert recommendations [1,2,3,11]. The following framework is not intended to replace individualized clinical judgment, but to structure the principles that should guide it.

### 10.1. Diagnosis and Clinical Characterization

Distinguishing paroxysmal TN from continuous trigeminal neuropathic pain, mixed facial pain, and other MS-related sensory syndromes is essential before any therapeutic decision [2,3,4,5]. High-resolution MRI should be obtained not only to confirm the demyelinating disease burden, but also to assess the presence of neurovascular conflict and to help classify the syndrome into three categories: (1) plaque-dominant, (2) compression-dominant, or (3) indeterminate [2,3,11,12,25,28]. These categories have operational value for guiding decision-making, but do not represent proven diagnoses, given the limited discriminative capacity of current neuroimaging.

### 10.2. Assessment of Baseline Vulnerability

Disability status, fatigue burden, gait stability, cognitive symptoms, and pharmacological treatment burden should be explicitly assessed. These factors condition both the tolerability of medical treatment and the risk–benefit balance of invasive procedures [6,7,8,9,10]. A patient who achieves partial pain suppression but becomes cognitively blunted or functionally unsafe on medication should be considered unsuccessfully treated, even if pain scores improve.

### 10.3. Medical Treatment as the First Step

Medical therapy remains the standard initial step, but should be continued only insofar as it remains tolerable and its impact on the patient’s function is acceptable [2,3,18,19]. Early consideration of surgical treatment is reasonable when medication toxicity becomes a major part of the problem.

### 10.4. Percutaneous Procedures and GKRS

These are appropriate options when the patient has substantial operative risk, indeterminate or plaque-dominant MRI findings, or expresses a preference for less invasive treatment [1,2,3,22,23]. They are supported by direct TN-MS literature, mainly for pain outcomes.

### 10.5. Microvascular Decompression

MVD may be considered in selected patients with acceptable operative risk, a predominantly classical, trigger-dominated clinical phenotype, and imaging evidence of neurovascular conflict. However, this recommendation is based on retrospective evidence and expert opinion, and the presence of neurovascular conflict alone does not guarantee favorable outcome. Additionally, in the absence of clearly demonstrated neurovascular conflict on preoperative imaging but in the presence of refractory pain despite multiple prior treatments and a clinical phenotype suggesting possible compressive pathology (paroxysmal character, trigger-dependence, clear dermatomal distribution), surgical exploration may still be warranted with the aims of identifying MRI-occult neurovascular conflicts intraoperatively or performing intraoperative neuromonitoring-guided techniques such as root combing and internal neurolysis, consistent with the dual-indication framework described in Section 8.

### 10.6. Defining Therapeutic Success

The assessment of any intervention should extend beyond pain-scale scores to include medication use, fatigue, cognitive tolerability, functional change, and sensory morbidity, although such endpoints have not yet been standardized in the literature [1,6,7,8,9,10].

## 11. Discussion

The central argument of this article is that the evidence on TN-MS treatment remains biased toward purely pain-based endpoints, and that this bias probably distorts decision-making and leads to over-selection, withholding potentially effective treatment modalities from many patients. This affects an already vulnerable population [6,7,8,9,10].

Pain resolution is the primary goal of any intervention, but it is methodologically insufficient as an isolated metric in the TN-MS setting. A patient with baseline neurological disability has a significantly lower tolerance for the neurotoxic effects of chronic pharmacological treatment—sedation, ataxia, cognitive dysfunction—than a neurologically intact individual with classical TN [6,7,8,9,10,26,27]. Consequently, the assessment systems that dominate the current literature, by omitting these functional variables, tend to overestimate the true clinical success of therapeutic strategies in the patient’s lived experience.

Polypharmacy deserves specific attention. The extensive MS literature shows that it is common and that a higher medication burden correlates with worse cognitive performance and lower quality of life [7,8,9,10]. Expert reviews on TN-MS consistently acknowledge that drug-related adverse effects frequently lead to an early invasive therapeutic approach [2,3]. These data strongly support two propositions: first, medication burden should be treated as a clinically meaningful outcome in TN-MS research; second, the absence of medication-burden reporting in surgical studies is itself an important evidence gap. The field often assumes that successful procedures reduce medication dependence and thereby improve function, but in TN-MS this has rarely been formally measured [1,2,3,11,22,23].

The same logic applies to quality of life. Pain is consistently associated with worse autonomy and health-related quality of life in patients with MS [6,7]. Although it is hypothetically plausible that better pain control and a lower medication burden would translate into improved quality of life in patients with TN-MS, the literature has not assessed this conclusively [1]. It is essential to state this gap openly rather than allowing indirect evidence to masquerade as direct support.

This conceptual framework helps to clarify the role of MVD. Its main appeal is that it represents the non-ablative therapeutic alternative with potentially more durable efficacy and a lower recurrence rate in a specific subgroup of patients with TN-MS, potentially reducing the effects of chronic pharmacological polytherapy [2,3,11,13,14,15]. In the absence of high-quality direct evidence, these conclusions rely largely on the interpretation of expert opinion and on the coherence of clinical reasoning. Indeed, the methodological debate open in the most recent literature—concerning whether outcome definitions and follow-up interpretation are too restrictive—illustrates precisely this tension [24,25]. This does not weaken the validity of the hypothesis, but rather defines the research agenda.

At our own institution, a small number of patients with MS-associated refractory TN (fewer than ten over the past decade) have undergone surgical exploration of the cerebellopontine angle, reflecting both the relative rarity of surgical referral for this condition and the caution with which it is generally approached. In our practice, when no neurovascular conflict is identified intraoperatively, root combing or internal neurolysis is performed rather than abandoning exploration without a therapeutic gesture. We consider this a reasonable approach in carefully selected patients, consistent with the dual-indication framework outlined in Section 8. Should a sufficiently large and well-characterized cohort become available, we intend to report it separately as a dedicated clinical study with prospectively defined, validated outcome measures.

Future studies must change both methodology and priorities. At a minimum, prospective TN-MS cohorts should record medication count, dose intensity, successful drug withdrawal, validated quality-of-life measures, fatigue scores, cognitive outcomes, sensory morbidity, and functional status, alongside conventional pain metrics [1,6,7,8,9,10]. Without such data, treatment recommendations will continue to be shaped by a literature that tends to oversimplify the therapeutic goal, eluding a far more complex clinical reality.

## 12. Conclusions

TN-MS should be understood as a pain syndrome superimposed on the neurological and therapeutic burdens intrinsic to MS. That context changes the meaning of treatment success [2,6,7,8,9,10].

Medical treatment remains the first-line standard of care, but it is based largely on extrapolation from classical TN outcomes and is often limited by tolerability in MS [1,2,3,18,19]. Percutaneous procedures and GKRS are reasonable options supported by retrospective TN-MS data, but recurrence is common and patient-centered outcomes are underreported [1,22,23]. Although MVD should not be systematically excluded on the basis of an MS diagnosis, its role in this clinical context remains selective and based on limited evidence [1,2,3,11,13,14,15,24,25].

Our own limited institutional experience with cerebellopontine angle exploration in this population, including the use of root combing or internal neurolysis when no neurovascular conflict is found, is consistent with this selective approach, but is not reported here as formal evidence given its small size; rare but serious posterior fossa risks (e.g., CSF leak, hearing loss, cranial neuropathy) must still be considered in patient counseling regardless of technique.

The essential, hitherto unresolved problem is that the evidence base remains structured around endpoints based almost exclusively on pain response, in a population that is more complex and frail than that of classical TN [6,7,8,9,10]. Redefining therapeutic success in TN-MS—incorporating medication burden, health-related quality of life, fatigue, and functional status—should be a priority of the research agenda.

## Figures and Tables

**Table 1:** Comparative assessment of treatment modalities in TN-MS.

Modality	Pain Outcomes (TN-MS)	Durability/Recurrence	Main Complications	Quality of Evidence	Evidence on Medication Burden/Quality of Life	Critical Appraisal
Carbamazepine/oxcarbazepine	First line; efficacy inferred by extrapolation from classical TN (indirect evidence) [1,2,3,18,19]	Limited by intolerance or incomplete efficacy [2,3]	Sedation, dizziness, hyponatremia, cognitive slowing [2,3,26,27]	Indirect for TN-MS	Scarce in TN-MS; the general MS literature suggests a negative impact of medication burden on cognition and health-related quality of life [7,8,9,10]	First-line standard, but frequently limited by tolerability in patients with MS; the cumulative burden of adverse effects should not be underestimated.
Percutaneous procedures	Short-term relief in retrospective series (low-quality direct evidence) [1,2,3]	Common recurrence; repeat procedures frequent [1,2,3]	Numbness, dysesthesia, corneal risk, masseter weakness [1,2,3]	Low; heterogeneous retrospective series	Medication reduction assumed, not measured in TN-MS [1]	Pragmatic option in patients with high operative risk; the cumulative sensory burden of repeat procedures may be underestimated in conventional reports.
GKRS	Significant relief in retrospective and institutional series and meta-analysis (low-to-moderate direct evidence) [1,22,23]	Lower durability than in classical TN; retreatment sometimes required [22,23]	Numbness, delayed response, sensory disturbance [22,23]	Low to moderate; retrospective	Health-related quality-of-life and medication-burden data scarce in TN-MS [1,22,23]	Reasonable non- invasive option; insufficiently studied from a patient-centered perspective in TN-MS.
MVD/surgical exploration	Benefit in selected retrospective series; pain-free rates lower than in classical TN, around 30% at long term in the available meta-analysis (low-quality direct evidence) [1,2,3,11,13,14,15,24,25,28]	Potentially more durable in selected cases; superiority not demonstrated, owing to selection bias [1,2,3,11,24]	Posterior fossa morbidity, CSF leak, hearing loss, cranial neuropathy [11,14,15]	Low; retrospective and highly selected	Value for medication reduction inferred, not demonstrated in TN-MS; restrictive outcome definitions may underestimate benefit [6,7,8,9,10,25]	Should neither be promoted generally nor systematically excluded; the indication is stronger with a convincing NVC on MRI; in the absence of NVC, consider only in highly selected cases [25,28].

Abbreviations: CSF, cerebrospinal fluid; GKRS, Gamma Knife radiosurgery; MVD, microvascular decompression; NVC, neurovascular conflict; TN, trigeminal neuralgia; TN-MS, trigeminal neuralgia associated with multiple sclerosis.

## Data Availability

Not applicable, as this critical narrative review does not report a primary research dataset.

## Abbreviations

BNI, Barrow Neurological Institute; CSF, cerebrospinal fluid; GKRS, Gamma Knife radiosurgery; HRQoL, health-related quality of life; MRI, magnetic resonance imaging; mRS, modified Rankin Scale; MS, multiple sclerosis; MVD, microvascular decompression; NVC, neurovascular conflict; PV, petrosal vein; REZ, root entry zone; RF, radiofrequency; TN, trigeminal neuralgia; TN-MS, trigeminal neuralgia associated with multiple sclerosis.
